# Addition of an affected family member to a previously ascertained autosomal recessive nonsyndromic hearing loss pedigree and systematic phenotype-genotype analysis of splice-site variants in *MYO15A*

**DOI:** 10.1186/s12920-022-01368-9

**Published:** 2022-11-18

**Authors:** Jin-Yuan Yang, Wei-Qian Wang, Ming-Yu Han, Sha-Sha Huang, Guo-Jian Wang, Yu Su, Jin-Cao Xu, Ying Fu, Dong-Yang Kang, Kun Yang, Xin Zhang, Xing Liu, Xue Gao, Yong-Yi Yuan, Pu Dai

**Affiliations:** 1 College of Otolaryngology Head and Neck Surgery, Chinese PLA General Hospital, Chinese PLA Medical School, 28 Fuxing Road, Beijing, 100853 People’s Republic of China; 2 National Clinical Research Center for Otolaryngologic Diseases, State Key Lab of Hearing Science, Ministry of Education, Beijing, People’s Republic of China; 3 Beijing Key Lab of Hearing Impairment Prevention and Treatment, Beijing, People’s Republic of China; 4 Department of Otolaryngology, PLA Rocket Force Characteristic Medical Center, 16# XinWai Da Jie, Beijing, 100088 People’s Republic of China; 5 Department of Otolaryngology, Head and Neck Surgery, Chinese PLA General Hospital Affiliated Hainan Hospital, Jianglin Road, Sanya, 572013 People’s Republic of China; 6 Hainan Province Clinical Research Center for Otolaryngologic and Head and Neck Diseases, Jianglin Road, Sanya, 572013 People’s Republic of China; 7 Department of Otorhinolaryngology, Qilu Hospital (Qingdao), Cheeloo College of Medicine, Shandong University, 758 Hefei Road, Qingdao, 266035 Shandong People’s Republic of China; 8 Postgraduate Training Base of Jinzhou Medical University, The PLA Rocket Force Characteristic Medical Center, 16# XinWai Da Jie, Beijing, 100088 People’s Republic of China

**Keywords:** Autosomal recessive sensorineural hearing loss, Pathogenicity, Splice-site variant, *MYO15A*

## Abstract

Pathogenic variants in *MYO15A* are known to cause autosomal recessive nonsyndromic hearing loss (ARNSHL), DFNB3. We have previously reported on one ARNSHL family including two affected siblings and identified *MYO15A* c.5964+3G > A and c.8375 T > C (p.Val2792Ala) as the possible deafness-causing variants. Eight year follow up identified one new affected individual in this family, who also showed congenital, severe to profound sensorineural hearing loss. By whole exome sequencing, we identified a new splice-site variant c.5531+1G > C (maternal allele), in a compound heterozygote with previously identified missense variant c.8375 T > C (p.Val2792Ala) (paternal allele) in *MYO15A* as the disease-causing variants. The new affected individual underwent unilateral cochlear implantation at the age of 1 year, and 5 year follow-up showed satisfactory speech and language outcomes. Our results further indicate that *MYO15A*-associated hearing loss is good candidates for cochlear implantation, which is in accordance with previous report. In light of our findings and review of the literatures, 58 splice-site variants in *MYO15A* are correlated with a severe deafness phenotype, composed of 46 canonical splice-site variants and 12 non-canonical splice-site variants.

**Supplementary Information:**

The online version contains supplementary material available at 10.1186/s12920-022-01368-9.

## Introduction

Hearing loss is one of the most common genetic sensory disorders, affecting one out of every 500–650 infants in the world [1]. Genetic factor accounts for approximately 50–60% of congenital sensorineural hearing loss cases [2]. It’s estimated that 70% of hereditary cases are nonsyndromic, meaning hearing loss is the only clinical manifestation. Hereditary hearing loss is extremely heterogeneous. To date, 124 deafness genes have been identified (http://hereditaryhearingloss.org/, updated 8/30/2021). The most prevalent type of hereditary hearing loss is autosomal recessive nonsyndromic hearing loss (ARNSHL), which accounts for about 80% of cases.

*MYO15A* (OMIM #602,666) variants have been shown to cause ARNSHL, DFNB3 (OMIM #600,316) in individuals from different populations worldwide [3]. In cochlea, myosin XVa, the protein encoded by *MYO15A*, is expressed at the tips of stereocilia in hair cells and plays as a motor protein that moves along actin filaments using energy from ATP hydrolysis. Transport of whirlin to the tips of the stereocilia by myosin XVa has been proved to be essential for the development and elongation of the stereocilia, which are essential for normal auditory function [4, 5]. In myosin XVa-deficient mice, no links between stereocilia were observed, implies that the mechano-transduction mechanism had been completely disrupted [6].

Previously, we reported on a family with two affected siblings who suffered severe to profound sensorineural hearing loss, DFNB3 [7]. Whole-exome sequencing (WES) of two affected siblings and unaffected parents was performed, and two compound heterozygous variants in *MYO15A* (NM_016239.4) were identified in the two affected siblings: c.8375 T > C (p.Val2792Ala) and c.5964+3G > A.

In the 8 year follow-up study, we identified one new affected individual in this family (III-1), who also showed congenital, profound sensorineural hearing loss, consistent with the DFNB3 phenotype. Bi-allelic variants in *MYO15A* were identified, including one novel splice-site variant c.5531+1G > C (maternal allele) and one previous identified missense variant c.8375 T > C (p.Val2792Ala) (paternal allele). In addition, an extensive genotype–phenotype correlation was conducted for *MYO15A* splice-site variants, which were filtered using the Professional edition of the Human Gene Mutation Database (HGMD) and summarized by a literature review.

## Materials and methods

### Subjects and clinical evaluations

The Chinese family of Han ethnicity with hearing loss reported here was followed up for 8 years after our initial report [7]. Medical history, temporal bone computed tomography (CT), otoscopy, pure tone audiometry (PTA) (for children under the age of six), auditory steady state response (for children under the age of six), acoustic immittance, auditory brainstem responses, and distortion product otoacoustic emission are all part of the clinical evaluation for hearing loss.

According to pure-tone audiometry (PTA) of the better ear, the average hearing threshold level at four air conduction frequencies (500, 1000, 2000, and 4000 Hz) was used to define the severity of hearing loss. According to the 2021 WHO classification of hearing loss, 20- < 35 dBHL was defined as mild, 35- < 50 dBHL was defined as moderate, 50- < 65dBHL was defined as moderate to severe, 65- < 80dBHL was defined as severe, and > 80 dBHL was defined as profound.

### Molecular analysis

WES genetic analysis was performed in two new affected individuals, including II-3 and III-1. A blood DNA extraction kit was used to extract genomic DNA from peripheral blood according to the manufacturer's instructions (TianGen, Beijing, China). DNA was sheared, ligated to adaptors, extracted, and ligation-mediated PCR was used to amplify it. For enrichment, a 1 μg DNA library was combined with Buffer BL and GenCap probe (MyGenostics, Beijing, China). The Illumina NovaSeq 6000 platform was used to load each collected library. The fraction of mapped reads was 97–99% and average depth was 100 bp. After filtering out low-quality and duplicate reads, clean data were aligned to the human reference genome hg19 using the Burrows-Wheeler Aligner. Variants were called using four types of software (SOAPsnp, GATK, Samtools, and Platypus) and annotated by ANNOVAR. Then, variants were associated with multiple databases, including gnomAD, Inhouse database (MyGenostics), with minor allele frequencies (MAF) < 0.05. To check the possible pathogenicity of candidate variants, SIFT, PolyPhen-2, MutationTaster, and GERP++ software were used. Trio-based bioinformatic analysis of WES data were used for recessive, dominant, and X-linked conditions. Manually classification of those variants was conducted based on American College of Medical Genetics and Genomics (ACMG)/Association for Molecular Pathology (AMP) guidelines for genetic hearing loss. Sanger sequencing was used to confirm potential pathogenic variants identified by these analyses. Primer sequences are provided in Additional file 1: Table S1. The sizes of PCR products are 654 bp (c.5531+1G > C), 652 bp (c.5964+3G > A) and 458 bp (c.8375 T > C).

### Literature review of genotype–phenotype correlation of MYO15A splice-site variants

An extensive genotype–phenotype correlation was conducted for *MYO15A* splice-site variants. The Human Gene Mutation Database (HGMD) Professional edition was used to screen the variants, which were then evaluated through a literature review.

### In silico* validation of splice-site variants*

To evaluate the splice site strength of different sequences, four prediction tools were used, including varSEAK (https://varseak.bio/), SpliceAI (https://github.com/Illumina/SpliceAI), CADD PHREAD (https://www.bio.tools/CADD_Phred#!), MaxEntScan (http://hollywood.mit.edu/burgelab/maxent/).

## Results

### Clinical findings

**Fig. 1:**
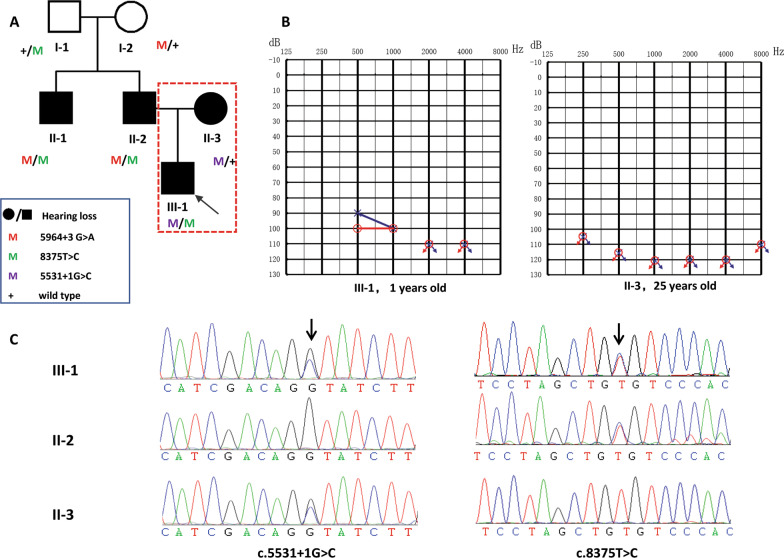
Extended family pedigree, hearing phenotype and variant analysis. **A** Affected individuals are denoted in black. The arrow indicates the proband. The red dashed line indicates the two new affected individuals; **B** Audiogram of the two new affected individuals showing profound sensorineural hearing loss (red, right ear; blue, left ear); **C** Chromatogram of *MYO15A* (NM_016239.4): c.5531+1G > C and c.8375C > T in three affected individuals (II-2, II-3, III-1)

A three-generation Chinese pedigree (Family 4794), depicted in Fig. 1A, expanded 2 samples (II-3 and III-1) from previous reported pedigree [7]. This family included 4 patients with hearing loss (II-1, II-2, II-3 and III-1). Among those four patients, the molecular basis of two affected sibling (II-1 and II-2) were identified as c.8375 T > C (p.Val2792Ala) and c.5964+3G > A in *MYO15A* in our previous study [7] and their hearing loss was congenital, bilateral, severe to profound, and sensorineural. For II-3, wife of II-2, her hearing loss was postlingual, late onset (8 years old), sensorineural and progressive. For III-1, his hearing loss was congenital, bilateral, severe to profound, and sensorineural. The Audiograms of the two new affected individuals (II-3 and III-1) were depicted in Fig. 1B. The onset age of II-3 is different from other three patients and is inconsistent with reported DFNB3 phenotype. In any of the affected people, gross motor development was not noticeably slowed. All of the participants' physical evaluations indicated no symptoms of systemic disease or dysmorphic characteristics. In II-3 and III-1, high-resolution CT of the temporal bone revealed no abnormalities, ruling out middle and inner ear anomalies.

### Genotyping

**Fig. 2:**
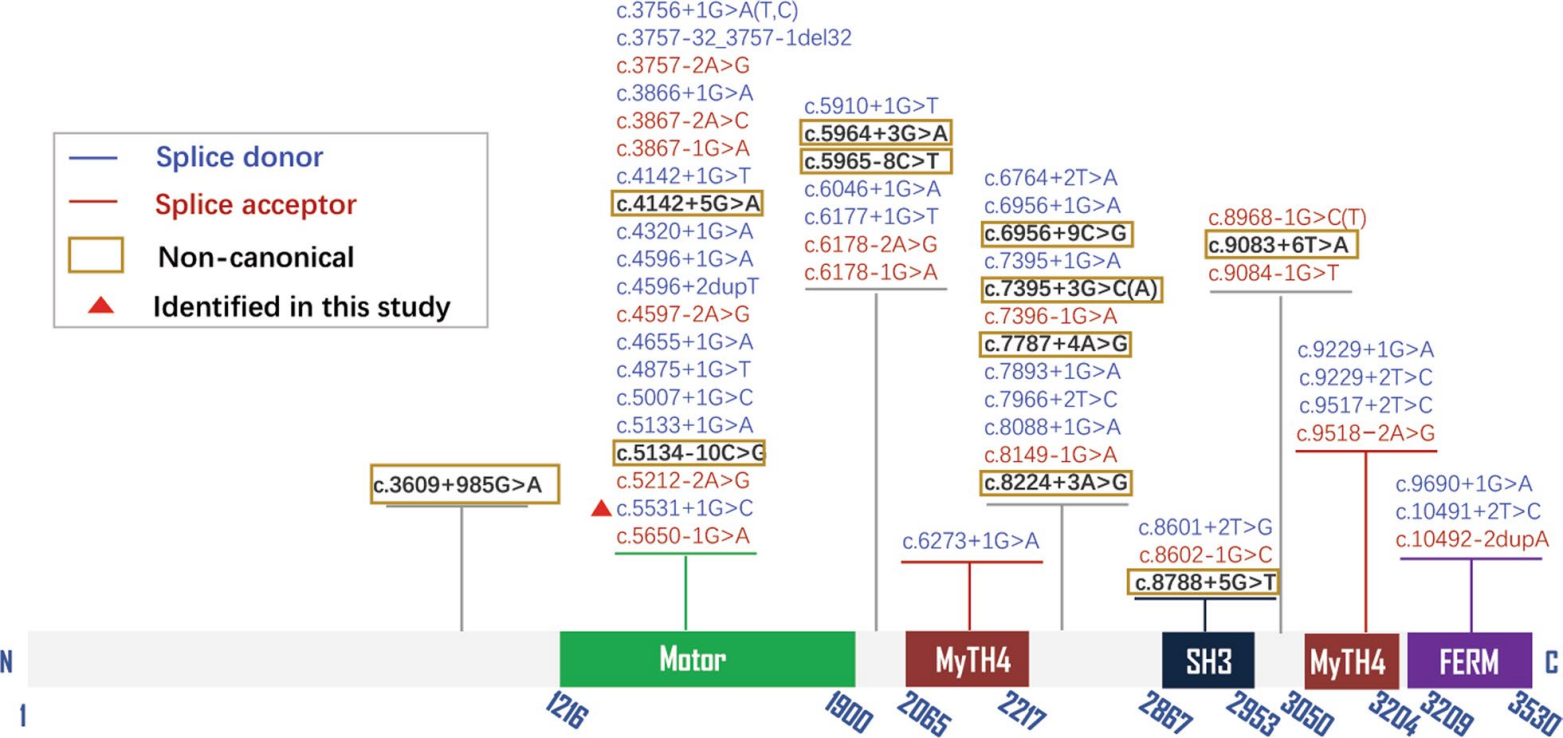
Locations of HGMD-reported splice-site variants in *MYO15A* (NM_016239.4)

The remaining variants were manually filtered based on their frequency/presence in known SNP databases, previous association with disease, predicted functional impact, nucleotide/amino acid conservation, and the potential detrimental biochemistry. The analysis identified compound heterozygous *MYO15A* variants c.5531+1G > C and c.8375 T > C (p.Val2792Ala) in III-1. There were no other potential variants in known deafness genes. Sanger sequencing was used to confirm the two discovered variants, and parental testing was used to validate them (Fig. 1C). The boy inherited the heterozygous c.5531+1G > C variant in *MYO15A* from his mother (II-3) and c.8375 T > C (p.Val2792Ala) from his father (II-2). No de novo or compound heterozygous variants in other deafness genes were identified in II-3 according to the autosomal dominant or recessive pattern of inheritance, as there is no maternal family history of hearing loss.

c.5531+1G > C variant is located in the intron region of the 5’ splice donor sequence and results from a G to A substitution (Table 1, Fig. 2). Several software programs including CADD PHREAD, varSEAK, and SpliceAI were used to evaluate the effect of the c.5531+1G > C variant on the splice site. Each analysis predicted that the substitution results in the loss of the donor site, causing altered splicing. According to ACMG/AMP guidelines, this variant is classified as pathogenic (PVS1+PM2+PM3+PP1+PP3).

**Table 1:** Summary of splice-site variants in *MYO15A* registered in HGMD

	Allele1					Allele2		
	Location	Nucleotide change	ACMG classification (codes)	MAF (gnomAD)	Zygosity	Location	Nucleotide change	AA change
1	Chr17:18,026,708	c.3609+985G > A	LP PS4+(PM2+PM3+PP1+PP3)	0	Hom			
					Het	Chr17:18,052,889	c.7207G > T	p.Asp2403Tyr
					Het	Chr17:18,065,953	c.9572G > A	p.Arg3191His
2	Chr17:18,028,546	c.3756+1G > A	P (PVS1+PP5+PM2+PP3)	0.00000802	Het	Chr17:18,039,881	c.4660G > A	p.Ala1554Thr
3	Chr17:18,028,546	c.3756+1G > T	P (PVS1+PM2+PP3+PP5)	0.0000318	Hom			
4	Chr17:18,028,546	c.3756++1G > C	P (PVS1+PM2+PP5+PP3)	0	Het	Chr17:18,052,097	c.6787G > A	p.Gly2263Ser
5	Chr17:18,029,626–18,029,658	c.3757-32_3757-1del32	P (PVS1+PM2+PP3)	0.00000808	NA			
6	Chr17:18,029,659	c.3757-2A > G	P (PVS1+PM2+PP3)	0	Hom			
7	Chr17:18,029,771	c.3866+1G > A	P (PVS1+PM2+PP5+PP3)	0.0000161	Hom			
					NA			
8	Chr17:18,030,103	c.3867-2A > C	P (PVS1+PM2++PP3)	0	NA			
9	Chr17:18,030,104	c.3867-1G > A	P (PVS1+PM2+PP3)	0	Het	Chr17:18,045,553	c.5810G > A	p.Arg1937His
10	Chr17:18,034,657	c.4142+1G > T	P (PVS1+PM2+PP3)	0	Hom			
11	Chr17:18,034,661	c.4142+5G > A	VUS (PM2+PP3)	0	Hom			
12	Chr17:18,035,881	c.4320+1G > A	P (PVS1+PM2+PP5+PP3)	0	Het	Chr17:18,049,349	c.6437G > A	p.Arg2146Gln
13	Chr17:18,039,139	c.4596+1G > A	P (PVS1+PM2+PP5+PP3	0.0000122	Hom			
					Het	Chr17:18,035,812	c.4252G > A	p.Gly1418Arg
14	Chr17:18,039,140	c.4596+2dupT	VUS (PM2+PP3)		Het	Chr17:18,045,553	c.5810G > A	p.Arg1937His
15	Chr17:18,039,729	c.4597-2A > G	P (PVS1+PM2+PP5+PP3)	0.00000803	Het	Chr17:18,057,199	c.8077del	p.Leu2693CysfsTer45
					Het	Chr17:18,077,164	c.10420A > G	p.Ser3474Gly
16	Chr17:18,039,790	c.4655+1G > A	P (PVS1+PM2+PP3)	0.0000201	NA	Chr17:18,051,884	c.6764+2 T > A	-
17	Chr17:18,040,994	c.4875+1G > T	P (PVS1+PM2+PP3)	0	Het	Chr17:18,030,390	c.3943G > A	p.Gly1315Arg
18	Chr17:18,041,561	c.5007+1G > C	P (PVS1+PM2+PP5+PP3)	0	Het	Chr17:18,047,111	c.6046+1G > A	-
19	Chr17:18,042,251	c.5133+1G > A	P (PVS1+PM2+PP5+PP3)	0	Het	Chr17:18,055,426	c.7894G > T	p.Val2632Leu
20	Chr17:18,042,838	c.5134-10C > G	VUS (PM2+BP4)	0	Het	Chr17:18,025,140	c.3026C > A	p.Pro1009His
21	Chr17:18,043,829	c.5212-2A > G	P (PVS1+PM2+PP5+PP3)	0	Het	Chr17:18,039,776	c.4642G > A	p.Ala1548Thr
22	Chr17:18,044,458	c.5531+1G > C	P (PVS1+PM2+PP5+PP3)	0	Het	Chr17:18,058,662	c.8375 T > C	p.Val2792Ala
23	Chr17:18,045,392	c.5650-1G > A	P (PVS1+PM2+PP3)	0.0000319	Hom			
24	Chr17:18,046,155	c.5910+1G > T	P (PVS1+PM2+PP3)	0	NA			
25	Chr17:18,046,936	c.5964+3G > A	LP (PM2+PM3+PP1+PP3+PP5)	0.0000287	Het	Chr17:18,058,662	c.8375 T > C	p.Val2792Ala
					Het	Chr17:18,060,348	c.8681_8682insA	p.His2895ThrfsTer31
					Het	Chr17:18,061,038	c.8791del	p.Trp2931GlyfsTer103
					NA			
26	Chr17:18,047,021	c.5965-8C > T	VUS (PM2+BP4)	0.00011	Het	Chr17:18,024,711	c.2597C > G	p.Ser866Trp
					Het	Chr17:18,024,532	c.2418C > T	p.Phe806 =
27	Chr17:18,047,111	c.6046+1G > A	P (PVS1+PM2+PP5+PP3)	0.0000261	Het	Chr17:18,041,561	c.5007+1G > C	
					Het			
28	Chr17:18,047,315	c.6177+1G > T	P (PVS1+PM2+PP3+PP5)	0	Het	Chr17:18,023,242	c.1128C > A	p.Tyr376Ter
					Het	Chr17:18,039,887	c.4666G > A	p.Ala1556Thr
					Het	Chr17:18,027,845	c.3658_3662del	p.Glu1221TrpfsTer23
					NA			
29	Chr17:18,047,809	c.6178-2A > G	P (PVS1+PP5+PM2+PP3)	0	Hom			
30	Chr17:18,047,810	c.6178-1G > A	P (PVS1+PM2+PP3+PP5)	0	Het	Chr17:18,022,844	c.730G > A	p.Asp244Asn
31	Chr17:18,047,907	c.6273+1G > A	P (PVS1+PM2+PP5+PP3)	0	Hom			
32	Chr17:18,051,884	c.6764+2 T > A	P (PVS1+PM2+PP3)	0.0000194	Het	Chr17:18,039,790	c.4655+1G > A	
					Het	Chr17:18,036,569	c.4351G > A	p.Asp1451Asn
					Het	Chr17:18,029,748	c.3844C > T	p.Arg1282Trp
					Het	Chr17:18,043,906	c.5287C > T	p.Arg1763Trp
33	Chr17:18,052,267	c.6956+1G > A	P (PVS1+PM2+PP3+PP5)	0.00000491	Het			
34	Chr17:18,052,275	c.6956+9C > G	VUS (PM2+PP3)	0.00000535	Het	Chr17:18,075,505	c.10251_10253del	p.Phe3420del
					Het	Chr17:18,049,252	c.6340G > A	p.Val2114Met
35	Chr17:18,054,080	c.7395+1G > A	P (PVS1+PM2+PP3)	0	NA			
36	Chr17:18,054,082	c.7395+3G > C	VUS (PM2+PP3)	0	Hom			
37	Chr17:18,054,082	c.7395+3G > A	VUS (PM2+PP3)	0.00000504	Hom			
38	Chr17:18,054,149	c.7396-1G > A	P (PVS1+PP5+PM2+PP3)	0.0000141	Het	Chr17:18,059,601	c.8552C > T	p.Ala2851Val
					Het	Chr17:18,058,523	c.8324G > A	p.Arg2775His
					Het	Chr17:18,040,941	c.4823C > A	p.Ala1608Glu
					Het	Chr17:18,045,435	c.5692C > T	p.Arg1898Ter
39	Chr17:18,054,845	c.7787+4A > G	VUS (PM2+PP3)	0	Hom			
40	Chr17:18,055,266	c.7893+1G > A	P (PVS1+PM2+PP3+PP5)	0.0000124	Het	Chr17:18,052,097	c.6787G > A	p.Gly2263Ser
					Het	Chr17:18,051,413	c.6580C > T	p.Arg2194Trp
41	Chr17:18,055,500	c.7966+2 T > C	P (PVS1+PP5+PM2+PP3)	0	Het	Chr17:18,034,837	c.4198G > A	p.Val1400Met
42	Chr17:18,057,211	c.8088+1G > A	P (PVS1+PM2+PP3+PP5)	0.00000402	NA			
43	Chr17:18,057,993	c.8149-1G > A	P (PVS1+PM2+PP5+PP3)	0	Hom			
44	Chr17:18,058,072	c.8224+3A > G	VUS (PM2+PP3)	0	Hom			
45	Chr17:18,059,652	c.8601+2 T > G	P (PVS1+PM2+PP5+PP3)	0	NA			
					NA			
46	Chr17:18,060,267	c.8602-1G > C	P (PVS1+PM2+PP3)	0	Het			
47	Chr17:18,060,549	c.8788+5G > T	VUS (PM2+PP3)	0	Hom			
48	Chr17:18,061,836	c.8968-1G > C	P (PVS1+PM2+PP3+PP5)	0	Hom			
49	Chr17:18,061,836	c.8968-1G > T	P (PVS1+PM2+PP5+PP3)	0.00000803	Het	Chr17:18,057,172	c.8050 T > C	p.Tyr2684His
					Hom			
50	Chr17:18,061,958	c.9083+6 T > A	LP (PS3+PM2+PP1+PP5+BP4)	0	Hom			
51	Chr17:18,062,238	c.9084-1G > T	P (PVS1+PM2+PP3+PP5)	0	NA			
52	Chr17:18,062,662	c.9229+1G > A	P (PVS1+PM2+PP3)	0	Hom			
53	Chr17:18,062,663	c.9229+2 T > C	P (PVS1+PM2+PP3+PP5)	0	Hom			
54	Chr17:18,064,763	c.9517+2 T > C	P (PVS1+PM2+PP5+PP3)	0.00000402	Het	Chr17:18,066,565	c.9620G > A	p.Arg3207His
55	Chr17:18,065,897	c.9518 − 2A > G	P (PVS1+PM2+PP3)	0.00000809	Hom			
56	Chr17:18,066,636	c.9690+1G > A	P (PVS1+PP5+PM2+PP3)	0	Hom			
					Hom			
57	Chr17:18,077,237	c.10491+2 T > C	P (PVS1+PM2+PP3+PP5)	0	Het	Chr17:18,023,248	c.1137del	p.Tyr380MetfsTer64
58	Chr17:18,082,081	c.10492-2dupA	P (PVS1+PM2+PP3)	0	Het	Chr17:18,057,446	c.8090 T > C	p.Val2697Ala

	Allele2		Hearing loss phenotype	Onset	Ethnicity	References
	ACMG classification (codes)	MAF (GnomAD)				
1			Severe to profound/moderate	Prelingual	Palestinian	Rayyan et al. [8]
	VUS (PM2+PP3+PP5)	0	Moderate	Prelingual	Palestinian	Rayyan et al. [8]
	LP (PM2+PM5+PP3+PP5)	0.0000401	Moderate to severe	Prelingual	Palestinian	Rayyan et al. [8]
2	VUS (PM2+PP3	0.0000722	Profound, Progressive	5yo	Japanese	Sakuma et al. [9]
3			Profound	Congenital	Pakistani	Liburd et al. [10]
4	LP (PM2+PM5+PP3)	0.0000309	Severe to profound, symm	Congenital	NA	Sloan-Heggen et al. [11]
5			NA	NA	Chinese(Taiwanese)	Wu et al. [12]
6			NA	Prelingual or congenital	Peruvian	Figueroa-Ildefonso et al. [13]
7			Severe to profound	Prelingual	Palestinian	Rayyan et al. [8]
			Severe to profound	NA	Pakistani	Nal et al. [14]
8			NA	NA	European(major)	Hou et al. [15]
9	VUS (PM2+PP3)	0.0000282	NA	NA	Iranian	Bazazzadegan et al. [16]
10			Severe to profound	Prelingual	Iranian	Sloan-Heggen et al. [17]
11			Severe to profound	Prelingual	Palestinian	Rayyan et al. [8]
12	LP (PM1+PM2+PP3+PP5)	0.0000121	Severe to profound	Diagnosed at an early age	Korean	Woo et al. [18]
13			NA	< 5yo	Iranian	Motavaf et al. [19]
	LP (PM2+PP5+PP3)	0.00000803	Profound	Congenital	Chinese	Zhang et al. [20]
14	VUS (PM2+PP3	0.0000282	Severe to profound, asymmetric	Childhood	NA	Sloan-Heggen et al. [11]
15	LP (PVS1++PM2)	0	Profound	Congenital	Chinese	Zhang et al. [20]
	LB (BP6+BP4+MP2)	0.000508				
16	P (PVS1+PM2+PP3)	0.0000194	NA	Congenital	NA	Sloan-Heggen et al. [17]
17	P (PVS1+PM2+PM5+PP3)	0	Profound	Congenital	Chinese	Liang et al. [21]
18	P (PVS1+PM2+PP5+PP3)	0.0000261	NA	Prelingual	European	Sommen et al. [22]
19	LP (PVS1+PM2)	0.00000647	NA	Congenital or prelingual	Turkish	Bademci et al. [23]
20	B (BS1+BS2+BP4)	0.00591	NA	NA	Chinese	Sun et al. [24]
21	VUS (PM2+PP3	0.0000201	Severe to profound	Congenital or prelingual	Turkish	Atik et al. [25]
22	VUS (PM2+PP3+PP5	0.00000401	Profound, symmetric	Congenital	Chinese	This study
23			NA	NA	Turkish	Duman et al. [26]
24			Severe to profound	Prelingual	Iranian	Sloan-Heggen et al. [17]
25	VUS (PM2+PP3+PP5	0.00000401	Severe to profound, symmetric	Prelingual	Chinese	Gao et al. [7]
	P (PVS1+PM2+PP3)	0	NA	NA	Chinese	Sun et al. [24]
	P (PVS1+PM2+PP3)	0	Profound	Congenital	Chinese	Zhang et al. [20]
			NA	NA	Chinese	Yang et al. [27]
26	VUS (PM2	0	Profound	Prelingual	Czech	Safka Brozkova et al. [28]
	LB (BP4+BP7+PM2)	0.000996				
27	P (PVS1+PM2+PP5+PP3)	0	NA	Prelingual	European	Sommen et al. [22]
			NA	Prelingual	European	Schrauwen et al. [29]
28	P (PVS1+PM2+PP3)	0	Severe to profound, symmetric	NA	Chinese	Sun et al. [30]
	VUS (PM2+PP3)	0.0000201	Severe, progressive	Prelingual(5yo)	Chinese	Zhang et al. [20]
	P (PVS1+PM2+PP3)	0.00000807	Profound, symmetric	Congenital	Chinese	Wang et al. [31]
			NA	NA	Chinese	Yuan et al. [32]
29			NA	NA	Pakistani	Rehman et al. [33]
30	VUS (PM2)	0.000392	NA	NA	Czech	Safka Brozkova et al. [28]
31			NA	Congenital or prelingual	Iranian	Yan et al. [34]
32	P (PVS1+PM2+PP3)	0.0000201	NA	Congenital	NA	Sloan-Heggen et al. [17]
	P (PM2+PP5+PP3)	0.000012	Profound	NA	Australian	Downie et al. [35]
	VUS (PM2)	0.0000563	NA	NA	Dutch	Zazo Seco et al. [36]
	VUS (PM2+PP3)	0.00177				
33			NA	NA	Chinese(Taiwanese)	Wu et al. [12]
34	VUS (PM2+PP3)	0.000016	NA	NA	Chinese	Yang et al. [27]
	LP (PM1+PM2+PP3+PP5)	0.00000402	NA	NA	Chinese	Yang et al. [27]
35			Severe to profound	Prelingual	Iranian	Sloan-Heggen et al. [17]
36			Severe to profound	NA	Tunisian	Belguith et al. [37]
37			Severe to profound	NA	Tunisian	Riahi et al. [38]
38	VUS (PM2+PP5+PP3)	0.000012	Severe to profound	NA	Vietnamese	Han et al. [39]
	VUS (PM2+PP3)	0.00000804	Severe to profound	NA	Vietnamese	Han et al. [39]
	VUS (PM2+PP3)	0	Profound	Congenital	Chinese	Zhang et al. [20]
	P (PVS1+PM2+PP5+PP3)	0.00000401	Severe	Congenital	Chinese	Zhang et al. [20]
39			NA	NA	Chinese	Yang et al. [27]
40	LP (PM2+PM5+PP3)	0.0000309	NA	NA	Dutch	Zazo Seco et al. [36]
	VUS (PM1+PM2)	0.000141	NA	Prelingual	European	Sommen et al. [22]
41	P (PP5+PM2+PP3)	0.0000361	NA	Congenital	NA	Sloan-Heggen et al. [11]
42			NA	NA	Chinese	Yuan et al. [32]
43			Profound	NA	N-African	Boudewyns et al. [40]
44			NA	NA	Pakistani	Rehman et al. [33]
45			Profound	Prelingual	Iranian	Sloan-Heggen et al. [17]
			Severe to profound	NA	Egyptian	Budde et al. [41]
46			NA	NA	Chinese(Taiwanese)	Wu et al. [12]
47			Severe to profound	Prelingual	Palestinian	Rayyan et al. [8]
48			Profound	Prelingual	Turkish	Kalay et al. [42]
49	VUS (PM2+PP5+PP3)	0.0000602	Severe to profound	Congenital	Spanish	Cabanillas et al. [43]
			Severe, stable, symmetric	Prelingual	NA	García-García et al. [44]
50			Profound	Congenital	Arab	Danial-Farran et al. [45]
51			NA	NA	Chinese	Yuan et al. [32]
52			Severe to profound	NA	Tunisian	Belguith et al. [37]
53			NA	NA	Pakistani	Rehman et al. [33]
54	VUS (PM2+PM5+PP3)	0.0000441	NA	Congenital	NA	Sloan-Heggen et al. [11]
55			Severe to profound	Congenital	Pakistani	Khan et al. [46]
56			Profound	Prelingual	Chinese(Uyghur)	Chen et al. [47]
			Profound, symmetric	Congenital	Chinese	Zhang et al. [48]
57	P (PVS1+PP5+PM2)	0.0000962	Severe	NA	Australian	Downie et al. [35]
58	P (PVS1+PM2+PP5+PP3)	0.000257	NA	Congenital or prelingual	Turkish	Yan et al. [34]

*N/A* Not available; *Het* Heterozygous; *Hom* Homozygous; *P* Pathogenic; *LP* Likely pathogenic; *VUS* Variants with uncertain significance; *B* Benign; *LB* Likely benign

### Cochlear implantation

Individual III-1 had been treated with unilateral cochlear implantation (Cochlear, Nucleus^®^ CI512) at the age of 1, and a 5 year follow-up demonstrated that his listening and language abilities had significantly improved, with a high degree of accuracy in speech perception and the development of near-normal language skills. Several studies have described the results of cochlear implantation in patients with DNFB3. Almost all reports suggested that cochlear implantation was satisfactory, similar with our case [49–52].

### Genotype–phenotype analysis of MYO15A reported splice-site variants

According to this study and HGMD Professional database (prior to Oct 1st, 2021), there were 360 DFNB3-associated pathogenic variants in *MYO15A*, including 58 splice-site variants that comprise a significant 16.11% (58/360) of pathogenic variants (Table 1, Fig. 2). We performed genotype–phenotype correlation analysis by literature review. The majority of pathogenic splice-site variants disrupt exons inside the Motor domain, which are believed to decrease Myosin VA protein function by affecting the capacity of whirlin transport to the tips of hair cell stereocilia. Pathogenic splice-site variants at Myosin Xavi’s N-terminal extension are less identified. The variants mainly distributed from Motor domain to FERM (protein 4.1-ezrin-radixin-moesin) functional domains (Fig. 2). Only one pathogenic splice-site variant in N-terminal was reported, c.3609+985 A > G, lies in intron 2. Although, variants in *MYO15A* lead to variable hearing impairment phenotype, from mild to severe, splice-site variants have been linked to a severe hearing loss phenotype in all identified cases, except those hearing loss degrees were not described in the literatures (Table 1).

### Assessment of pathogenicity to non-canonical splice-site variants

Among the 58 considered disease-causing splice-site variants, 46 were canonical that in general change the + 1, + 2, − 2 and − 1 residue of an intron, and the remaining 12 were presumably non-canonical splice-site variants, accounting for 20.69% (Table 2). As for 46 canonical splice-site variants in *MYO15A*, the number of donor and acceptor splice site variants was 30 (65.22%) and 16 (34.78%), respectively (Fig. 2). The remaining 16 were splice site variants and account for 34.78% (Fig. 2). Eight out of 12 non-canonical splice-site variants were absent in GnomAD (Table 1). Although c.5134-10C > G, c.5965-8C > T, c.7787+4A > G, c.8224+3A > G, c.8788+5G > T, were registered in HGMD as pathogenic variants, their interpretations of pathogenicity are conflicting. Only 1 sporadic patient was reported to be associated with these variants. These variants were classified as variants of unknown significance, according to ACMG/AMP guidelines, and their association with disease necessitated further investigation.

**Table 2:** Overview of non-canonical splice-site variants in *MYO15A*

	Variant	Predicted effect on pre-mRNA splicing	Number of patients		In silico prediction					Minigene splicing assay	Zygosity	ACMG classification (Code)
				CADD PHREAD	varSEAK Online	SpliceAI	MaxEntScan alt	MaxEntScan diff	MaxEntScan ret			
1	c.3609+985G > A	Cryptic exon inclusion	8	18.5	Class 1: no splicing effect	Donor Loss: 0.27	N/A	N/A	N/A	No	Het/ Hom	LP PS4+(PM2+PM3+PP1+PP3)
2	c.4142+5G > A	Exon Skipping	2	25.7	"Class 5: splicing effect (Loss of function for authentic Splice Site. Exon Skipping)	Donor Loss: 0.90	2.649	6.604	9.253	No	Hom	VUS (PM2+PP3)
3	c.5134-10C > G	No splicing effect	1	4.72	Class 1: no splicing effect	0	11.18	1.105	12.288	No	Het	VUS (PM2+BP4)
4	c.5964+3G > A	Exon Skipping	4	9.911	Class 2: likely no splicing effect	Donor Gain:0.34	9.65	-2.577	7.075	No	Het	LP (PM2+PM3+PP1+PP3+PP5)
5	c.5965-8C > T	No splicing effect	1	2.737	Class 1: no splicing effect	Acceptor Gain:0.02	9.05	0.868	9.917	No	Het	VUS (PM2+BP4)
6	c.6956+9C > G	Create ectopic splice site	2	22.4	"class 5: splicing effect (Use of a cryptic site 4 nt downstream of 3' ss	Donor Loss:0.66	N/A	N/A	N/A	No	Het	VUS (PM2+PP3)
7	c.7395+3G > C	Create a cryptic splice donor site	1	15.82	Pos 6956+5: Strong increase of Score. New Splice Site.)"	Donor Loss:0.26	6.60	3.495	10.098	No	Hom	VUS (PM2+PP3)
8	c.7395+3G > A	Exon skipping	1	11.53	"Class 5: splicing effect (Loss of function for authentic Splice Site. Exon Skipping	Acceptor Gain/Donor Gain:0.01	10.65	-0.548	10.098	No	Hom	VUS (PM2+PP3)
9	c.7787+4A > G	Exon skipping	1	14.42	"Class 4: likely splicing effect (Likely loss of function for authentic Splice Site. Exon Skipping	Donor Loss:0.15	6.32	0.841	7.162	No	Hom	VUS (PM2+PP3)
10	c.8224+3A > G	Exon Skipping	1	23.3	Pos 7787 + 1: Decrease of Score for authentic Splice Site.)"	Donor Loss:0.79	-4.99	5.665	0.674	No	Hom	VUS (PM2+PP3)
11	c.8788+5G > T	Exon Skipping	1	22.8	"Class 4: likely splicing effect (Likely loss of function for authentic Splice Site. Exon Skipping	Donor Loss:0.82	-3.85	7.146	3.292	No	Hom	VUS (PM2+PP3)
12	c.9083+6 T > A	Exon Skipping	1	23.9	Pos 8224 + 1: Decrease of Score for authentic Splice Site.)"	Donor Loss:0.71	3.73	4.554	8.281	Yes	Hom	LP (PS3+PM2+PP1+PP5+BP4)

*N/A* Not available; *Het* Heterozygous; *Hom* Homozygous; CADD scores greater than 20 are considered to be pathogenic; *P* Pathogenic; *LP* Likely pathogenic; *VUS* Variants with uncertain significance

**Fig. 3:**
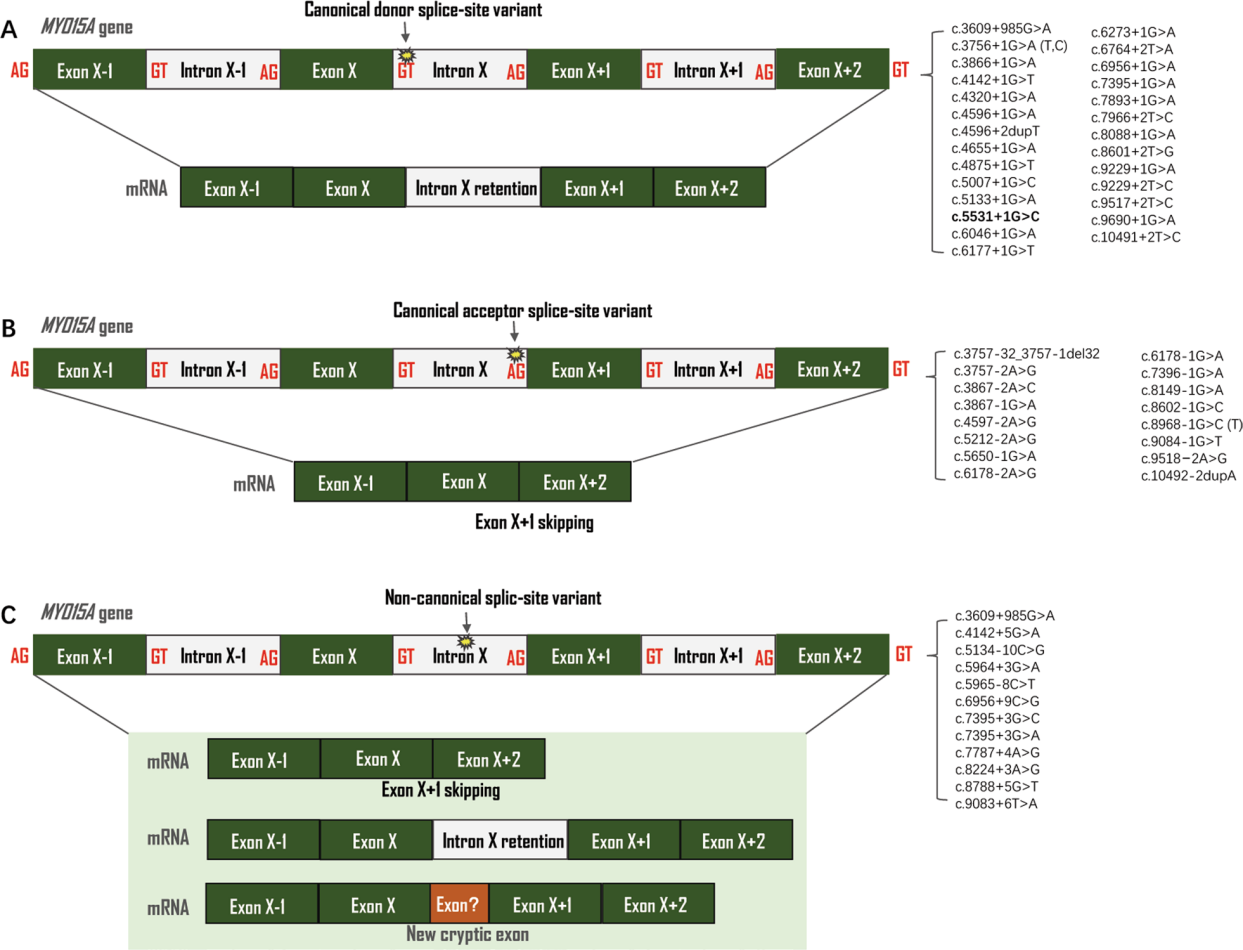
The types of *MYO15A* reported splice-site variants. Green boxes are exons and white boxes are introns. A yellow asterisk indicates the site of variant. **A** Canonical donor splice-site variant leads to intro retention; **B** Canonical acceptor splice-site variant leads to exon skipping; **C** Non-canonical splice-site variant. Deep intronic variants creating new splice sites

Figure 3 summarizes the results of the 58 splice-site variants in *MYO15A* that predict to produce a great variety of splicing outcomes. Variants that destroy natural donor sequences seem to cause the skipping of their associated exon while variants in acceptor sequence are associated with intron retention. It should be noted that in a considerable number of cases, additional events can also take place. Pathogenic non-canonical mRNA alterations, which are normally associated with common events like intron retention or selective exon skipping, can also include cryptic events that occur outside of conventionally designated exons and unconventional splicing processes that regulate gene expression.

## Discussion and conclusion

Since our initial report of *MYO15A* variants as the ARNSHL-associated gene among individuals with hearing loss in the Chinese population in 2013, several pathogenic variants of this gene have been identified in case–control studies with Chinese participants [53, 48, 54]. Our recent study of 511 Chinese individuals with hearing loss identified a genetic spectrum and showed that the disease-causing variants in *MYO15A* were the third most common cause (0.92%) of ARNSHL, behind *GJB2* and *SLC26A4* variants [32].

The variant c.5531+1G > C in *MYO15A* has never been reported in cases with hearing loss and was not presented in the public database. c.5531+1G > C occurs in *trans* with the reported pathogenic variant c.8375 T > C in *MYO15A*. It is well known that individuals with *MYO15A-*associated hearing loss (DFNB3) often present with nonsyndromic, congenital, severe to profound sensorineural hearing loss with normal middle and inner ear structure. Given the fact that II-3’s hearing loss was late-onset and progressive, which is atypical of DFNB3, it is possible that other genes or other factors are responsible for II-3’s hearing loss. Although the etiology of the II-3 hearing loss was not confirmed, there is a at least 50% chance the couple’s (II-2 and II-3) children will have *MYO15A*-associated hearing loss, as she is a heterozygous variant carrier of *MYO15A*. Pre-implantation genetic testing may be used to assess the risk for hearing loss.

*MYO15A* contains 67 exons and allows for a wide range of transcriptional variability, with the longest mRNA transcript being 3,530 amino acids. It encodes a N-terminal extension domain, ATPase motor domain, two myosin-tail homology 4 (MyTH4) domains, a Src-homology-3 (SH3) domain, and a band 4.1 superfamily (FERM) domain (Fig. 2).

According to this study and HGMD professional database, 360 pathogenic variants of *MYO15A* have been identified. According to a recent study, 27% of splicing variations linked to severe dominant developmental disorders are not found inside the canonical splice site [55], which is similar to 20.69% obtained in this study. The most common *MYO15A* mutation type is missense alteration in the exonic region. Nonsense, in-frame deletion, splice-site variations, intragenic deletions and duplications are less common forms [12]. Between introns 2–65, 58 identified splice-site variations have been reported, accounting for 16.11% (58/360) of pathogenic variants in *MYO15A* (Table 1).

Spliceosomes are responsible for pre-mRNA splicing in humans [56]. The donor splice-site variants were more common than the acceptor splice-site variants, according to the literature review (ratio 1.5:1). We have observed that, in *MYO15A*, splice-site variants affect the 5' splice donor site (70.59%) more frequently than the 3ʹ donor site (29.41%).

Normal pre-mRNA splicing that define exon–intron boundaries at the + 1, + 2, − 2 and − 1 residue of an intron is usually disrupted by these canonical splice-site variants, and lead to the development of a slew of hereditary diseases [57]. However, because these intronic cis-elements are not always highly conserved and their modifications do not always impair the splicing processes, it is unclear whether non-canonical splice-site variants would result in RNA-splicing errors [58]. They may yield new cryptic exons as well as splice variants in retained intron. Despite the fact that c.3609+985A > G is positioned deep within intron 2 (more than 100 base pairs away from exon–intron boundaries), several lines of evidence suggest that it has a negative impact on the gene product. This mutation was projected to result in the loss of this putative exon’s donor site. The variant cosegregated with hearing loss in at least 8 Palestinian ARNSHL families and was not present in any public database. The reference base pair was conserved among multiple species. The 150-bp genomic sequence immediately proximal to the variant site was predicted to have exotics potential based on conservation analysis. It is predicted that c.3609+985A > G leads to the loss of this hypothetical exon’s donor site [8].

It is accessible to acquire *MYO15A* RNA from patients’ inner ear to assess the effect of variants on expression directly. Multiple in silico prediction computer algorithms have been developed to predict the results of non-canonical splice-site variants [59, 60]. Due to the high complexion of splicing regulation, in silico prediction methods lack sufficient specificity and sensitivity for reliable application. By combining the outputs of multiple predictive tools, a more accurate prediction can be achieved. However, such in silico tools, even for combination, can only be used as a single piece of integrated supporting evidence in the evaluation of pathogenicity [55, 61]. The in vitro minigene splicing assay provides a useful tool for analysis of splice events, including RT-PCR, cell-based minigene assays, and massive parallel reporter assays [61]. A transient minigene experiment for c.9083+6 T > A revealed the abnormal splicing pattern, which could be caused by disruption of U1 snRNP binding to the 5ʹ splice-site, which prevents splicing initiation and results in exon 52 skipping [45, 62]. The Human Splicing Finder program predicted that c.6956+9C > G would result in a strong ectopic splicing site (HSF score of 80.6) [27]. In order to provide a better understanding of alternative tissue-specific splicing mechanism, in vivo minigene assay have been applied in the zebrafish and *C. elegans* [63, 64]. It’s not completely understood how some splice-site variants disrupt normal translation and produce unusual transcriptional products in the inner ear. The precise medical care for DFNB3 patients will benefit from a better understanding of mRNA processing from mutant *MYO15A*.

## Web resources

varSEAK, https://varseak.bio/. SpliceAI, https://github.com/Illumina/SpliceAI. CADD, https://www.bio.tools/CADD_Phred#!. MaxEntScan, http://hollywood.mit.edu/burgelab/maxent/.

## Supplementary Information

**Additional file 1: Table S1.** Primers sequences.

## Data Availability

The patients’ phenotype and detected variants were submitted to ClinVar (https://www.ncbi.nlm.nih.gov/clinvar/) under the accession numbers SCV001332616.1, SCV001332617.1, and SCV001332618.1.

## Abbreviations

ARNSHL: Autosomal recessive nonsyndromic hearing loss
WES: Whole exome sequencing
ACMG: American college of medical genetics and genomics
AMP: Association for molecular pathology
