# Supplementary material for: Addition of an affected family member to a previously ascertained autosomal recessive nonsyndromic hearing loss pedigree and systematic phenotype-genotype analysis of splice-site variants in MYO15A
Source: BMC Med Genomics. 2022 Nov 18;15:241. doi: 10.1186/s12920-022-01368-9 (PMC9673454; doi:10.1186/s12920-022-01368-9)
Supplement: Supplementary file 1 — Additional file 1: Table S1. Primers sequences. [file 12920_2022_1368_MOESM1_ESM.docx]

**Supplemental Table 1 Primers sequences**

|  | **Forward primer** | **Reverse primer** |
| --- | --- | --- |
| c.8375T>C | GAACCAGCTGGACACACAGA | AAATGGGTTTGCTTCAATGG |
| c.5964+3G>A | GCCTCTGTTTCTCTCTTGCTCT | GAAGTGGCACTGGACAAACTTG |
| c.5531+1G>C | AGGTGCAACCCCTTGTTCAT | ACAGGACACTTGACTGCCAC |
